# Clinical Outcomes of Genotype-Matched Therapy for Recurrent Gynecological Cancers: A Single Institutional Experience

**DOI:** 10.3390/healthcare9101395

**Published:** 2021-10-19

**Authors:** Kiyoka Sawada, Kentaro Nakayama, Kohei Nakamura, Yuki Yoshimura, Sultana Razia, Masako Ishikawa, Hitomi Yamashita, Tomoka Ishibashi, Seiya Sato, Satoru Kyo

**Affiliations:** Department of Obstetrics and Gynecology, Shimane University School of Medicine, Izumo 693-8501, Japan; kiyoka.0504@gmail.com (K.S.); knakamura320@keio.jp (K.N.); y-yuki@med.shimane-u.ac.jp (Y.Y.); raeedahmed@yahoo.com (S.R.); m-ishi@med.shimane-u.ac.jp (M.I.); memedasudasu1103@gmail.com (H.Y.); tomoka@med.shimane-u.ac.jp (T.I.); sato_seiya9534@yahoo.co.jp (S.S.); satoruky@med.shimane-u.ac.jp (S.K.)

**Keywords:** gynecological cancer, clinical sequence, genotype-matched therapy

## Abstract

Recent advances in next-generation sequencing and genome medicine have contributed to treatment decisions in patients with cancer. Most advanced gynecological cancers develop resistance to chemotherapy and have a poor prognosis. Therefore, we conducted genomic tests in gynecological tumors to examine the efficacy and clinical feasibility of genotype-matched therapy. Target sequencing was performed in 20 cases of gynecological cancers (cervical cancer, 6; endometrial cancer, 6; and ovarian cancer, 6). Both actionable and druggable genes were identified in 95% (19/20) of the cases. Among them, seven patients (35%) received genotype-matched therapy, which was effective in three patients. Of the three patients, one patient with a *PTEN* mutation received everolimus, another patient with a *TSC2* mutation received everolimus and letrozole, and the patient with a *BRIP1* mutation received olaparib. Subsequently, disease control in these three patients lasted for more than half a year. However, all patients relapsed between 9 and 13 months after the initiation of genotype-matched therapy. In this study, the response rate of genotype-matched therapy was 43% (3/7), which may have contributed to improved prognoses. Therefore, genotype-matched therapies may help patients with refractory gynecological cancers achieve better outcomes.

## 1. Introduction

A total of 41,000 women are newly diagnosed with gynecological cancer in Japan each year, and approximately 10,000 women die from gynecological cancers [1]. The current standard treatment for gynecological cancer includes surgery, cytotoxic chemotherapy, and radiotherapy. These treatment methods are performed alone or in combination, depending on the stage of the disease and the patient’s clinical situation. For advanced gynecological cancers, first-line chemotherapy or adjuvant chemotherapy is performed; however, currently available cytotoxic chemotherapeutic drugs are limited, and the prognosis is often poor despite the use of chemotherapy. Furthermore, in ovarian cancer, recurrent tumors are often resistant to chemotherapy compared to the initial treatment [2]. Therefore, more effective treatments for gynecological cancers are needed.

In recent years, with the development of molecular biology techniques, targeted therapies against various molecular targets have been developed. For example, poly (ADP-ribose) polymerase (PARP) inhibitors for *BRCA1/2* mutation-positive cases in ovarian cancer and programmed cell death protein 1 (PD-1) monoclonal antibody for microsatellite instability (MSI-high) in solid tumors. In a phase 2 study, it was reported that using PD-1 monoclonal antibody such as pembrolizumab for MSI-high solid tumors excluding intestinal cancer has been effective [3].

With the advent of next-generation sequencing (NGS), it has become possible to identify gene alterations related to carcinogenesis in each patient and to comprehensively analyze cancer genomes. Furthermore, NGS has been applied both clinically and in research. Genotyped-matched therapy based on the results of the NGS test has been initiated in patients with cancer; however, the use of NGS is still limited.

There are reports that targeted therapies for patient tumors using NGS have better outcomes than therapies that did not use NGS [4,5]; however, there is not enough evidence on improved prognoses or methods of target treatment selection. This study aimed to analyze the molecular characteristics of gynecological cancers that were treated at our institution, and the effect and clinical usefulness of genotype-matched therapy. We investigated whether the NGS test is reliable and useful for treatment selection of patients with gynecological cancer using a 160-gene panel test.

## 2. Results

### 2.1. Patient’s Clinical Information and Genome Sequence

From January 2018 to January 2019, genomic tests were performed on 20 patients with recurrent gynecological cancer. The patient characteristics are listed in Table 1. Among 20 cases, there were eight cases of cervical cancer (CC), six cases of endometrial cancer (EC), and six cases of ovarian cancer (OC). The median age was 58.5 years (range, 39–75). The median number of actionable gene alterations per patient was three (range, 0–9), and the median number of prior therapies was two (range, 0–4). The most common actionable gene alteration was *TP53* (7/20 patients analyzed, 35%), followed by *PTEN* (4/20, 20%) and *PIK3CA* (4/20, 20%). The other actionable gene alterations were less than three. Both actionable gene alteration and druggable gene alteration rates were 95% (19/20 cases). Targeted therapy was performed in 35% of the cases (7/20), and the response rate (rate of stable disease [SD] or partial response [PR] or complete response [CR]) was 43% (3/7 cases) (Table 1).

### 2.2. Mutation Map

**Figure 1:**
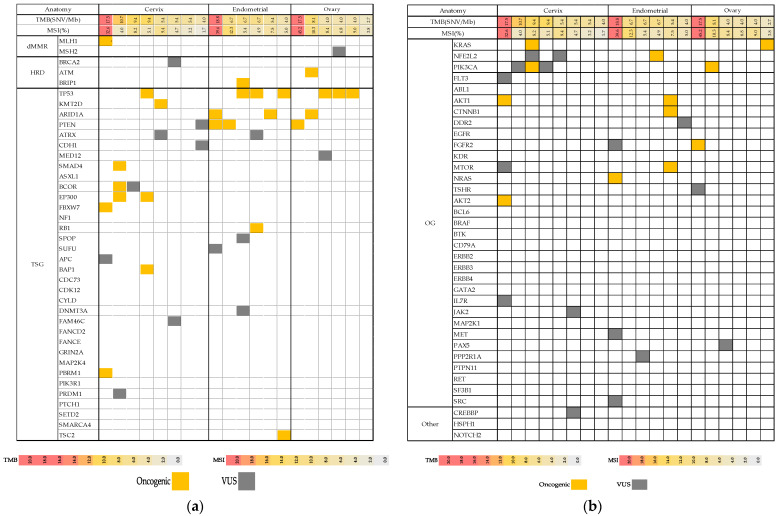

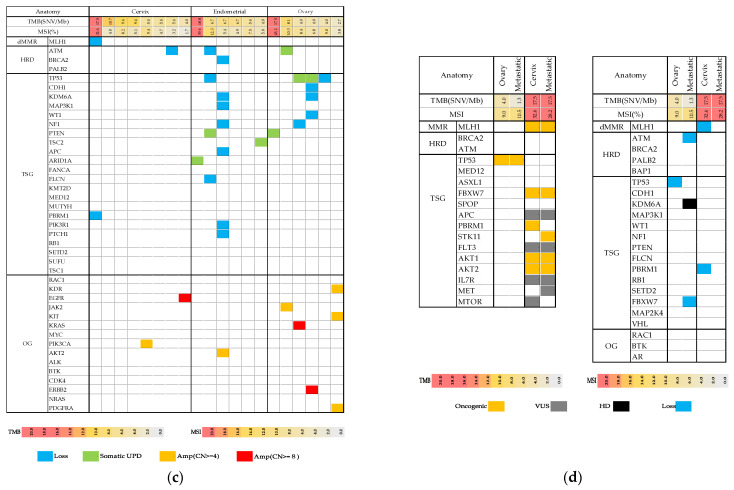
Distribution of molecular alterations identified by 160-gene panel investigation. (**a**). Genes related to dMMR, HRD, and TSG alterations. Orange is an oncogenic alteration and gray is VUS. (**b**). There are OG alterations and others identified by 160-gene panel investigations. Orange is an oncogenic alteration and gray is VUS. (**c**). There are various types of molecular alterations: blue, loss; green, somatic UPD; orange, amp (CN > 4); and red, amp (CN > 8). (**d**). Comparison of gene mutations in primary and metastatic lesions in ovarian and cervical cancers.TMB: tumor mutation burden, MSI: microsatellite instability, dMMR: deficient mismatch repair, HRD: homologous recombination deficiency, TSG: tumor suppressor gene, VUS: variants of unknown significance, OG: oncogene, UPD: uniparental disomy, Amp: amplification.

Genomic testing of 20 patients identified 70 molecular alterations. Among the gene alterations, 35 tumor suppressor gene (TSG), 28 oncogene (OG), four homologous recombination deficiency (HRD)-related gene, two deficient mismatch repair (dMMR)-related gene, and one other gene alterations were observed (Figure 1a,b).

Seventy molecular alterations included 18 deletions, seven somatic uniparental disomy (UPD), and seven amplifications (CNV > 4 = 5, CNV > 8 = 2) (Figure 1c). Comparison of gene alterations between the primary and metastatic lesions in one ovarian cancer and one cervical cancer cases showed almost similar distribution between primary and metastatic lesions (Figure 1d). Both cases showed similar actionable variant patterns; however, the deletion patterns were different between the primary and metastatic sites.

### 2.3. Genotype-Matched Therapy

Genotype-matched therapy was performed in seven cases (35%). Among them, there was one case of cervical cancer, five cases of endometrial cancer, and one case of ovarian cancer. Pembrolizumab was used in two patients (based on MSI-high status): one patient had progressive disease (PD), and the other had an unknown outcome for transfer to another hospital. Olaparib was used in one patient (based on HRD) who showed a CR on a computed tomography (CT) scan, which was performed after 2 months. However, 9 months later, recurrence was observed. Four patients were treated with everolimus (based on *PTEN*, *TSC2*, *mTOR*, *CTNNB1*, and *NF1* status): in one case (based on *PTEN* status), only everolimus was used, whereas in the rest of the cases it was combined with letrozole.

The patients with EC who only used everolimus were positive for the neuron specific enolase (NSE) marker in immunohistochemistry. In Japan, in most cases, the cost of genotype-matched therapy is not covered by insurance. If the patients are positive for the NSE marker, insurance can be used to cover their expenses. For this reason, letrozole was not used. One study found that a combination of everolimus and letrozole was associated with extend PFS in cases of ER-positive high-grade ovarian cancer in a phase Ⅱ study [6]. Therefore, in our study, a combination of everolimus and letrozole was used in cases of ovarian cancer if the patient was positive for ER. One case (based on *PTEN* status) showed SD, one case (based on *TSC2* status) showed PR, and two cases had PD (Table 2).

### 2.4. Three Cases of Successful Genotyped-Matched Therapy

#### 2.4.1. Case 1

**Figure 2:**
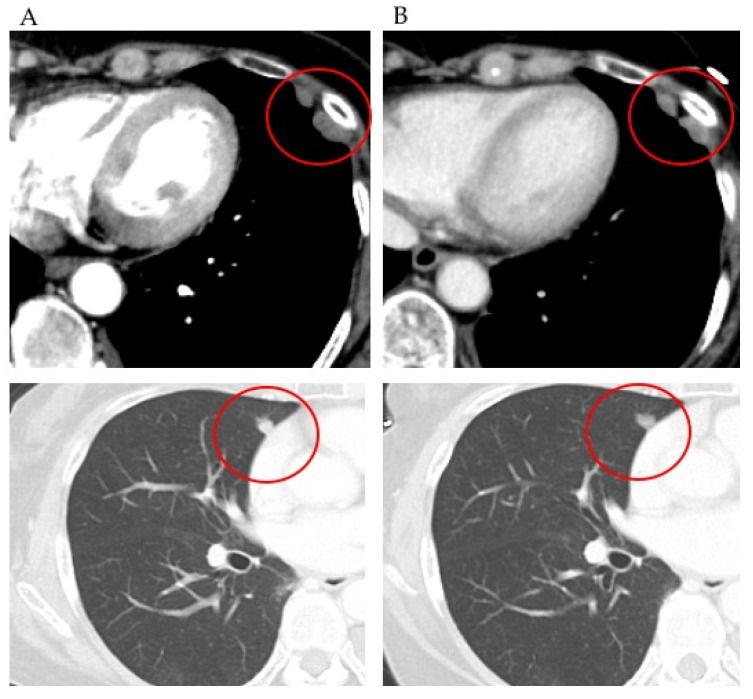
(**A**) Chest computed tomography showing a lung metastatic lesion before everolimus treatment. (**B**) Chest computed tomography showing a shrunken lung metastatic lesion, treated with everolimus, at the end of the first month.

A 65-year-old woman who was diagnosed with stage IB endometrial cancer, G3 endometrioid carcinoma after surgery, received six cycles of carboplatin and paclitaxel. Twenty-three months after chemotherapy, multiple lung metastases were found on a CT scan and after that genomic test was performed. The patient received six cycles of doxorubicin and cisplatin. However, 5 months later, the number of multiple lung metastases increased and pleural dissemination appeared. The patient was initially treated with everolimus (based on a *PTEN* mutation) 9 months after the genomic test. After 1 month, these lesions were stable (Figure 2) and continued to remain so until 13 months after the initiation of everolimus therapy, after which the lesions were found to be increased on a CT scan.

#### 2.4.2. Case 2

**Figure 3:**
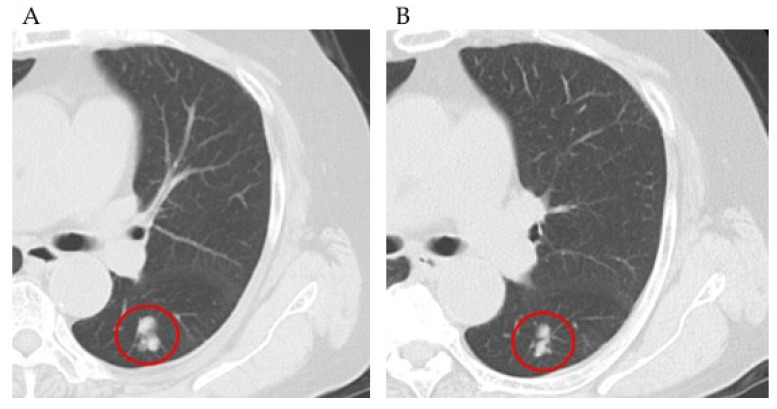
(**A**) Chest computed tomography showing a lung metastatic lesion before everolimus treatment. (**B**) Chest computed tomography showing a lung metastatic lesion after treatment with everolimus, end of 1 month, with decreased size.

A 68-year-old woman diagnosed with stage IVB endometrial cancer (multiple lung metastases), G3 endometrioid carcinoma underwent six cycles of carboplatin and paclitaxel after surgery. Nineteen months after chemotherapy, multiple lung metastases were observed on a CT scan and then a genomic test was performed. The patient received six cycles of doxorubicin and cisplatin. However, 3 months later, the number of multiple lung metastases increased. Furthermore, thrombocytopenia appeared. The patient was started on everolimus 10 months after the genomic test, and everolimus therapy was ceased for 3 weeks. After platelet recovery, everolimus dose was reduced to half and the therapy was re-initiated. However, 10 months after the initiation of everolimus therapy, multiple lung metastases were found to be increased on a CT scan (Figure 3).

#### 2.4.3. Case 3

A 70-year-old woman was diagnosed with stage IVB endometrial cancer (multiple swollen cardiophrenic, paraaortic, and left lateral iliac lymph nodule, omental cake), G3 endometrioid carcinoma. The patient underwent six cycles of carboplatin and paclitaxel after surgery. The genomic test was performed prior to chemotherapy. Details of this patient’s clinical sequence and therapy have already been reported in our previous case report [7]. Therefore, this case is briefly introduced in the present report. After chemotherapy, SD was observed on a CT scan. The patient received six cycles of doxorubicin and cisplatin. Subsequently, the lesions were stable with swollen paraaortic and left lateral iliac lymph nodules. The patient was then administered with PARPi olaparib (based on HRD and *BRIP1* mutation status) 8 months after the genomic test. Two months after the initiation of olaparib therapy, all swollen lymph nodules disappeared as observed on a CT scan. However, 9 months after starting olaparib therapy, tumor recurrence was indicated on a CT scan.

## 3. Discussion

In this study, a panel test of 160 cancer-related genes was conducted, which revealed that both actionable and druggable gene alterations were found in 95% of the cases (19/20). The most frequent actionable gene mutation was *TP53* (35%), followed by *PTEN* (20%), and *PIK3CA* (20%). It has been reported that *PIK3CA* is more frequently found in solid tumors, such as breast cancer and lung cancer [8]. In gynecological cancers, it has also been reported that gene alterations of *PTEN* and *PIK3CA* are more frequent [9]. *PTEN* mutations were found in 50% of endometrial cancer [10]. In other reports of endometrial cancer, *PIK3CA* was detected in 22% of cases [11] and Makker et al. [12] reported it to be in 24% of cases. The results for the detected gene alterations in this study were similar to those reported previously.

In addition, in this study, we compared the differences in gene mutations between the primary and metastatic lesions in two cases. However, almost similar distributions of genetic alterations were observed in either case, and the actionable mutations were similar. The current finding is similar to those in previous reports, which state that there is almost no difference in actionable mutations between the primary and metastatic lesions [8].

In this study, druggable gene alterations were found in 95% of the cases. The SHIVA trial reported that gene alteration that could lead to treatment was found in 40% of cases [13] and another study [14] reported that clinically actionable genes were detected in 37% of cases, while the MOSCATO 01 trial reported it to be in 49.3% of cases [15]. Compared with previous reports, more druggable gene alterations were detected in the current study.

However, in reality, only 35% of the patients (7/20) received genotype-matched therapy. A study so far conducted in Japan reported that targeted therapy was performed in 13.3% [16], and another study [17] reported that it was performed in 15.2% of the patients. Thus, the patients who were able to receive targeted therapy were limited. The main reasons for failure included worsening condition before treatment and lack of participation in clinical trials. In addition, the availability of off-label drugs depended on the patient’s financial status. All of these issues have been highlighted in similar reports [18,19]; therefore, it is important to conduct genomic tests earlier and determine the available drugs or clinical trials before the patient’s condition deteriorates.

Of the seven patients who received genotype-matched therapy, five had endometrial cancer, one had cervical cancer, and one had ovarian cancer. Of these patients, only three patients with endometrial cancer (43%) achieved the effectiveness of genotype-matched therapy. These three successful patients remained without any deterioration in patient condition for more than half a year; however, all cases recurred within 9–13 months after the initiation of genotype-matched therapy. Some previous trials have evaluated the effectiveness of genotype-matched therapy in patients with difficult-to-treat cancers. In one report on advanced gastrointestinal cancer, the disease control rate was 45% [20]. In another report on metastatic breast cancer, 56% of the patients experienced clinical benefits with a progression-free survival ratio ≥ 1.3 [21]. The MOSCATO 01 trial [15] showed improved outcomes in 33% of patients with advanced cancers. In the SAFIR01 trial [22], 30% of the patients had an objective response or SD. In the SHIVA randomized trial [13] when comparing genotype-matched therapy and conventional standard therapy, no significant difference was found in progression-free survival. In contrast, according to another report, genotype-matched therapy had a better outcome than unmatched therapy [18].

Patients who received targeted therapy had better outcomes than those who did not. One patient with *PTEN* mutation received the mTOR inhibitor, i.e., everolimus treatment, the second patient with *TSC2* mutation also received everolimus and an aromatase inhibitor, letrozole. *PTEN* and *TSC2* genes are related to the phosphoinositol-3 kinase (PI3K)/AKT/mammalian target of rapamycin (mTOR) pathway. This pathway is often dysregulated in gynecological cancers [23] and is associated with an aggressive disease and a poor prognosis [24]. Therefore, mTOR inhibitors may provide a novel therapeutic approach for the dysregulation of the PI3K/AKT/mTOR pathway. The effectiveness of a single-agent therapy with mTOR inhibitors in recurrent metastatic EC has been reported [11]. Furthermore, recently, letrozole in combination with everolimus showed a high clinical benefit rate and response rate in a phase II clinical trial in advanced EC [25]. In this study, mTOR inhibitors were effective in both patients, therefore, we expect that mTOR inhibitors could improve prognosis in recurrent EC with dysregulation of the PI3K/AKT/mTOR pathway. However, in advanced gynecological cancers, which exhibit standard treatment resistance, the effect of PI3K pathway inhibitors is not due to mutations in the PI3K pathway [9]. Further investigation of the effect of mTOR inhibitors is warranted.

In this study, we showed that genomic testing will be useful in advanced recurrent gynecological cancer to identify new treatment options based on gene alterations. The limitation of this study is the small sample size. To overcome this limitation, strategies are being devised to increase the number of patients for clinical sequencing in our hospital. Furthermore, genotype-matched therapy may help improve gynecological cancer outcomes. Recently, cancer genomic tests have become available for the health insurance system in Japan, and whole exome analysis at one’s own expense has also been investigated. Therefore, more genomic alterations are expected to be revealed for larger sample sizes. Another limitation was that we used cancer tissues obtained by surgery or biopsy at first onset. We did not check whether any genomic changes occurred during chemotherapy. Nowadays, new noninvasive approaches, such as cell-free DNA (cfDNA) analysis, have been attracting more attention. Therefore, at the time of recurrence, it may be essential to check for treatment-induced genotype changes using cfDNA.

In summary, we performed clinical sequencing in 20 cases of gynecological cancers and found that the response rate of genotype-matched therapy was 43% (3/7), which may contribute to improved prognoses. Therefore, genotype-matched therapies may help patients with refractory gynecological cancers achieve better outcomes.

## 4. Materials and Methods

### 4.1. Study Design and Patients

We performed a target sequence to examine mutations in 160 cancer-related genes in patients with advanced gynecological cancer (*n* = 20). Participants were aged ≥20 years, had previously received at least one chemotherapy, had a performance status ≤1, and had a prognosis of at least 3 months or more. Acquisition of tumor tissues was approved by the Shimane University Institutional Review Board (IRB No. 20070305-1 and No. 201701207-1, Approval date: 5 March 2007, 26 June 2018). The study was conducted in accordance with the tenets of the Declaration of Helsinki and Title 45 (United States Code of Federal Regulations), Part 46 (Protection of Human Subjects), effective from December 13, 2001. All participants provided informed consent.

### 4.2. Genomic Analysis

Genomic tests were performed using a PleSSision^®^ internal clinical sequencing apparatus (Keio University, Tokyo, Japan). After obtaining informed consent for genomic testing, including tumor biopsy and analysis of germline DNA, we prepared samples from cancer tissues and cut them into 10 μm sections. The sample and peripheral blood extracted from patients with cancer were sent to the Keio University. Genomic DNA was extracted from tumor samples and peripheral blood.

To check the quality of the extracted DNA, the DNA integrity number (DIN) was calculated using an Agilent 2000 TapeStation (Agilent Technologies, Waldbronn, Germany). Next, the targeted amplicon exome sequencing analysis of the 160 genes was conducted using the Illumina MiSeq sequencing platform (Illumina, San Diego, CA, USA). The 160 genes investigated in this study are listed in Supplementary Figure S1.

The sequencing data were input to the Genome Jack bioinformatics pipeline (Mitsubishi Space Software, Tokyo, Japan) for analysis. Cancer-specific changes in somatic genes, including single-base variants (SNVs), insertions/deletions, and copy number alterations (CNVs), have been detected and compared with published data to investigate the clinical significance of these variants. Furthermore, secondary germline findings were identified by comparing the genomic profiles obtained from the tumor samples and peripheral blood. The sequence report created in this manner was returned to our institution for approximately 3 weeks, after which analysis at the cancer genomic board (CGB) was performed.

### 4.3. Definition of Actionable and Druggable Variants

We used ‘‘Clinical Practice Guidance for Next-generation Sequencing in Cancer Diagnosis and Treatment (Edition 1.0)’’ as definition of actionable and druggable gene variants [26]. Details of defining actionable and druggable variants have already been reported in previous report [27]. Supplementary Figure S2 shows details of categories of drug recommendation levels.

#### CGB

Based on the report obtained from the results of genomic tests, we had discussions at the CGB at the Keio University and other related hospitals, consisting of medical oncologists, pathologists, and medical geneticists. In this report, the actionable (cancer-related gene), druggable (related to the effectiveness of treatment), and germline gene variants were presented. On the basis of these variants, a comprehensive treatment strategy considering the clinical background such as the treatment history of patients and the availability of treatment insurance was recommended. However, few targeted therapies include clinical trials or self-pay care fees. Thus, the recommended treatment could not be provided in all cases.

## Figures and Tables

**Table 1:** Patient characteristics (*n* = 20).

Age (years)	
Median (range)	58.5 (39–75)
Tumor type, *n* (%)	
cervical cancer	8 (40%)
endometrial cancer	6 (30%)
ovarian cancer	6 (30%)
Genomic alterations, *n* (%)	
*TP53*	7 (35%)
*PTEN*	4 (20%)
*PIK3CA*	4 (20%)
Actionable alteration, *n* (%)	19 (95%)
Druggable alteration, *n* (%)	19 (95%)
Prior chemotherapy (*n*)	
Median (range)	2 (0–4)
Genotype matched therapy	
Matched therapy, *n* (%)	7 (35%)
Response rate, *n* (%)	3/7 (43%)

**Table 2:** Summary of patients who received genotype-matched therapy.

No	Age	Stage	Tumor Type	Histological Type	Targets	Targeted Therapy	Best Response
1	62	IIB	CC	SCC	Hypermutated and MSI-high MLH1 (Loss)	pembrolizumab	PD
2	65	IB	EC	EM(G3)	PTEN (mut)	everolimus	SD
3	68	IVB	EC	EM(G3)	TSC2 (somatic UPD)	everolimus letrozole	PR
4	59	IVB	EC	EM(G3)	AKT1, MTOR, CTNNB1(mut)	everolimus letrozole	PD
5	59	IVB	EC	EM(G3)	Hypermutated and MSI-high	pembrolizumab	-
6	70	IVB	EC	HGSC	HRD, BRIP1(mut)	olaparib	CR
7	41	IVB	OC	HGSC	NF1 (Loss)	Everolimus letrozole	PD

## Data Availability

Data of the current study are available on request from the corresponding author (K.N.)

## Supplementary Materials

The following are available online at https://www.mdpi.com/article/10.3390/healthcare9101395/s1, Figure S1: Comprehensive Cancer Panel: 160 gene list, Figure S2: Criteria and categories of genotype-matched therapy based on clinical sequencing.
