# Supplementary material for: Clinical Outcomes of Genotype-Matched Therapy for Recurrent Gynecological Cancers: A Single Institutional Experience
Source: Healthcare (Basel). 2021 Oct 19;9(10):1395. doi: 10.3390/healthcare9101395 (PMC8535840; doi:10.3390/healthcare9101395)
Supplement: Supplementary file 1 [file healthcare-09-01395-s001.zip › healthcare-1390564-supplementary.pptx]

## Slide 1
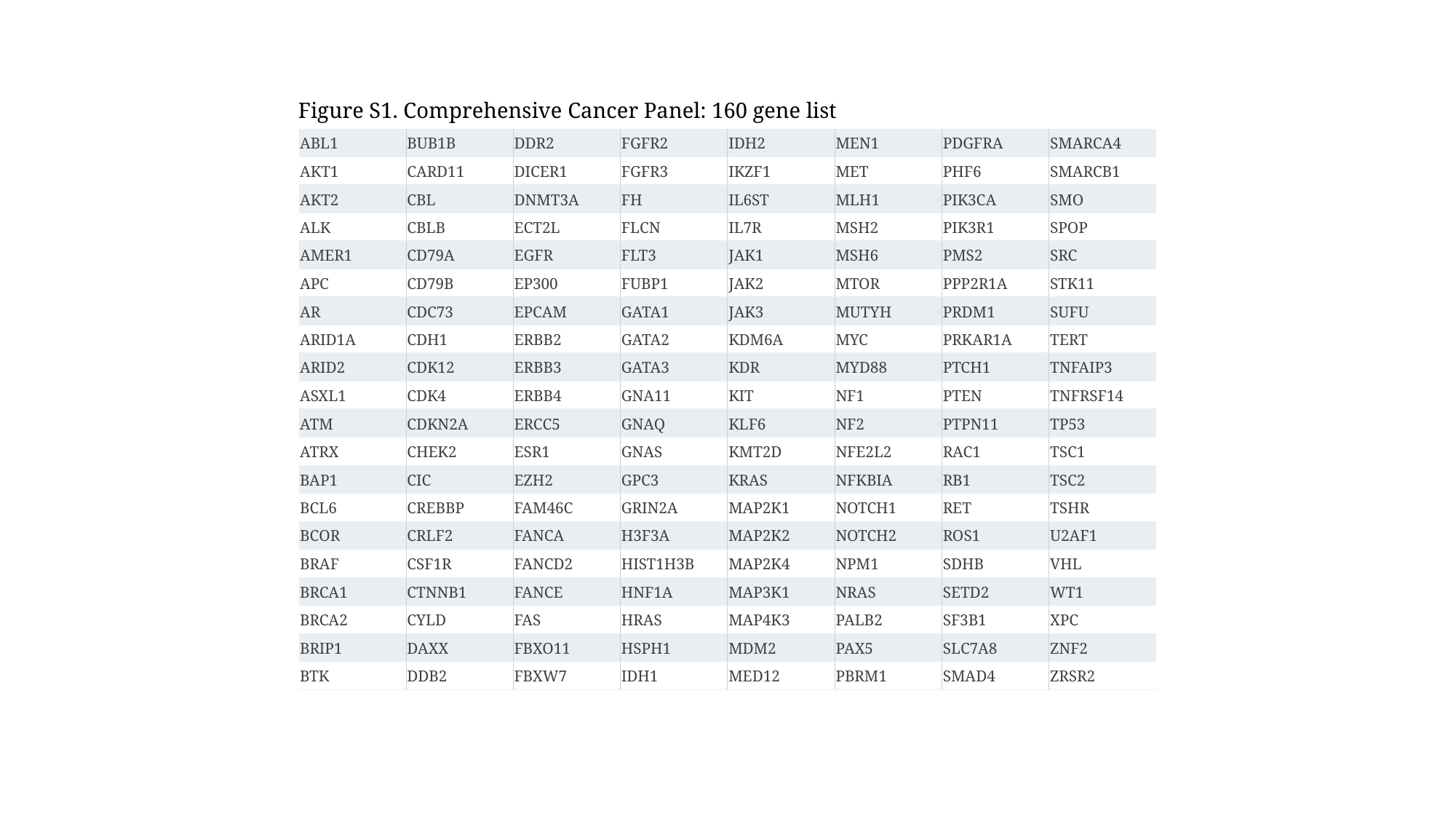

Figure S1. Comprehensive Cancer Panel: 160 gene list
| ABL1 | BUB1B | DDR2 | FGFR2 | IDH2 | MEN1 | PDGFRA | SMARCA4 |
| --- | --- | --- | --- | --- | --- | --- | --- |
| AKT1 | CARD11 | DICER1 | FGFR3 | IKZF1 | MET | PHF6 | SMARCB1 |
| AKT2 | CBL | DNMT3A | FH | IL6ST | MLH1 | PIK3CA | SMO |
| ALK | CBLB | ECT2L | FLCN | IL7R | MSH2 | PIK3R1 | SPOP |
| AMER1 | CD79A | EGFR | FLT3 | JAK1 | MSH6 | PMS2 | SRC |
| APC | CD79B | EP300 | FUBP1 | JAK2 | MTOR | PPP2R1A | STK11 |
| AR | CDC73 | EPCAM | GATA1 | JAK3 | MUTYH | PRDM1 | SUFU |
| ARID1A | CDH1 | ERBB2 | GATA2 | KDM6A | MYC | PRKAR1A | TERT |
| ARID2 | CDK12 | ERBB3 | GATA3 | KDR | MYD88 | PTCH1 | TNFAIP3 |
| ASXL1 | CDK4 | ERBB4 | GNA11 | KIT | NF1 | PTEN | TNFRSF14 |
| ATM | CDKN2A | ERCC5 | GNAQ | KLF6 | NF2 | PTPN11 | TP53 |
| ATRX | CHEK2 | ESR1 | GNAS | KMT2D | NFE2L2 | RAC1 | TSC1 |
| BAP1 | CIC | EZH2 | GPC3 | KRAS | NFKBIA | RB1 | TSC2 |
| BCL6 | CREBBP | FAM46C | GRIN2A | MAP2K1 | NOTCH1 | RET | TSHR |
| BCOR | CRLF2 | FANCA | H3F3A | MAP2K2 | NOTCH2 | ROS1 | U2AF1 |
| BRAF | CSF1R | FANCD2 | HIST1H3B | MAP2K4 | NPM1 | SDHB | VHL |
| BRCA1 | CTNNB1 | FANCE | HNF1A | MAP3K1 | NRAS | SETD2 | WT1 |
| BRCA2 | CYLD | FAS | HRAS | MAP4K3 | PALB2 | SF3B1 | XPC |
| BRIP1 | DAXX | FBXO11 | HSPH1 | MDM2 | PAX5 | SLC7A8 | ZNF2 |
| BTK | DDB2 | FBXW7 | IDH1 | MED12 | PBRM1 | SMAD4 | ZRSR2 |

## Slide 2
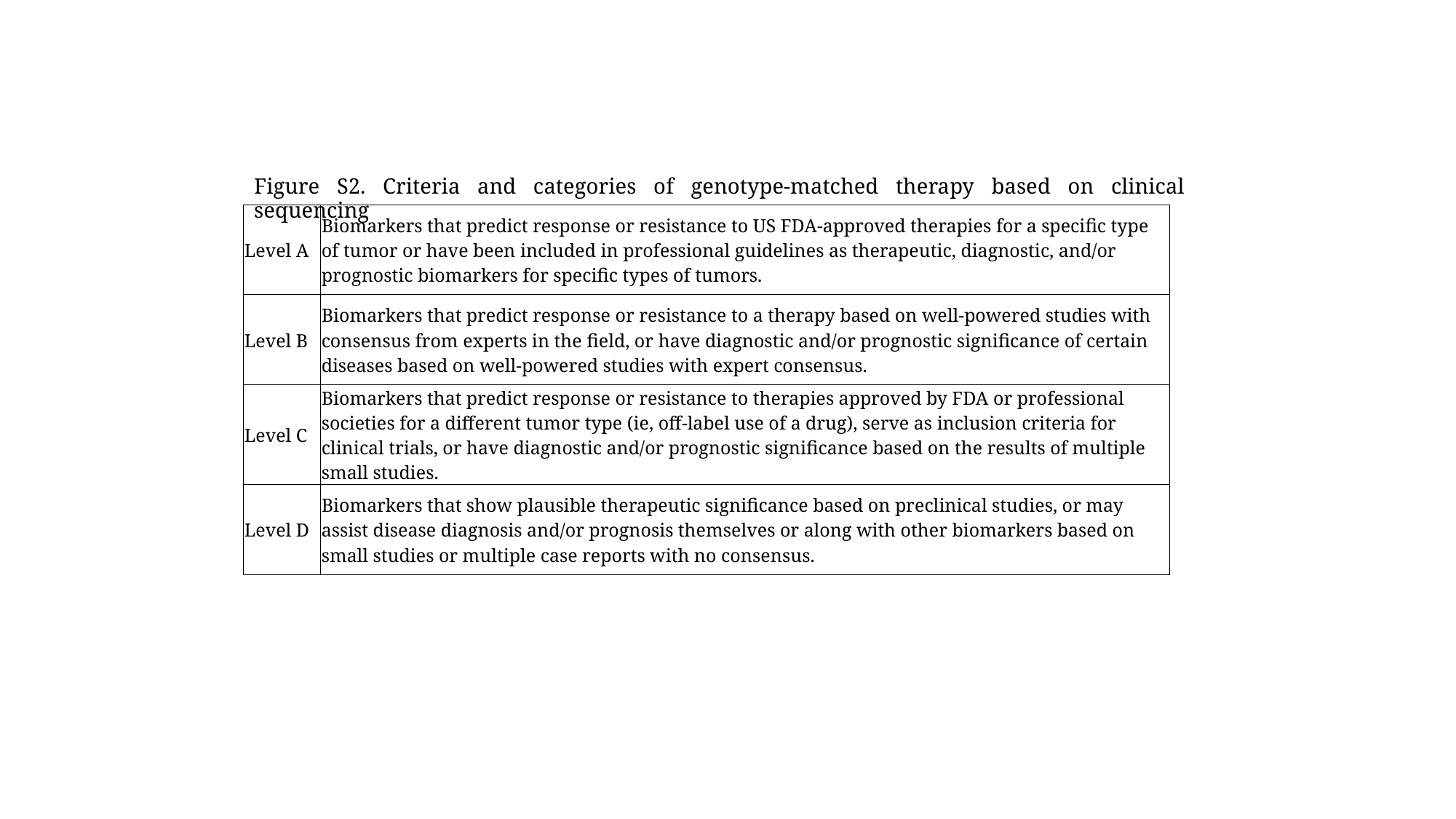

Figure S2. Criteria and categories of genotype-matched therapy based on clinical sequencing
| Level A | Biomarkers that predict response or resistance to US FDA‐approved therapies for a specific type of tumor or have been included in professional guidelines as therapeutic, diagnostic, and/or prognostic biomarkers for specific types of tumors. |
| --- | --- |
| Level B | Biomarkers that predict response or resistance to a therapy based on well‐powered studies with consensus from experts in the field, or have diagnostic and/or prognostic significance of certain diseases based on well‐powered studies with expert consensus. |
| Level C | Biomarkers that predict response or resistance to therapies approved by FDA or professional societies for a different tumor type (ie, off‐label use of a drug), serve as inclusion criteria for clinical trials, or have diagnostic and/or prognostic significance based on the results of multiple small studies. |
| Level D | Biomarkers that show plausible therapeutic significance based on preclinical studies, or may assist disease diagnosis and/or prognosis themselves or along with other biomarkers based on small studies or multiple case reports with no consensus. |
